# The temporal behavior of the murine T cell receptor repertoire following Immunotherapy

**DOI:** 10.1038/s41597-023-01982-x

**Published:** 2023-02-23

**Authors:** Tom Snir, Hagit Philip, Miri Gordin, Alona Zilberberg, Sol Efroni

**Affiliations:** The Mina & Everard Goodman Faculty of Life Sciences, Bar-Ilan University, Ramat-Gan, Israel

**Keywords:** VDJ recombination, Clonal selection, VDJ recombination, Predictive medicine

## Abstract

Immunotherapy is now an essential tool for cancer treatment, and the unique features of an individual’s T cell receptor repertoire are known to play a key role in its effectiveness. The repertoire, famously vast due to a cascade of cellular mechanisms, can be quantified using repertoire sequencing. In this study, we sampled the repertoire over several time points following treatment with anti-CTLA-4, in a syngeniec mouse model for colorectal cancer, generating a longitudinal dataset of T cell clones and their abundance. The dynamics of the repertoire can be observed in response to treatment over a period of four weeks, as clonal expansion of specific clones ascends and descends. The data made available here can be used to determine treatment and predict its effect, while also providing a unique look at the behavior of the immune system over time.

## Background & Summary

**Fig. 1:**
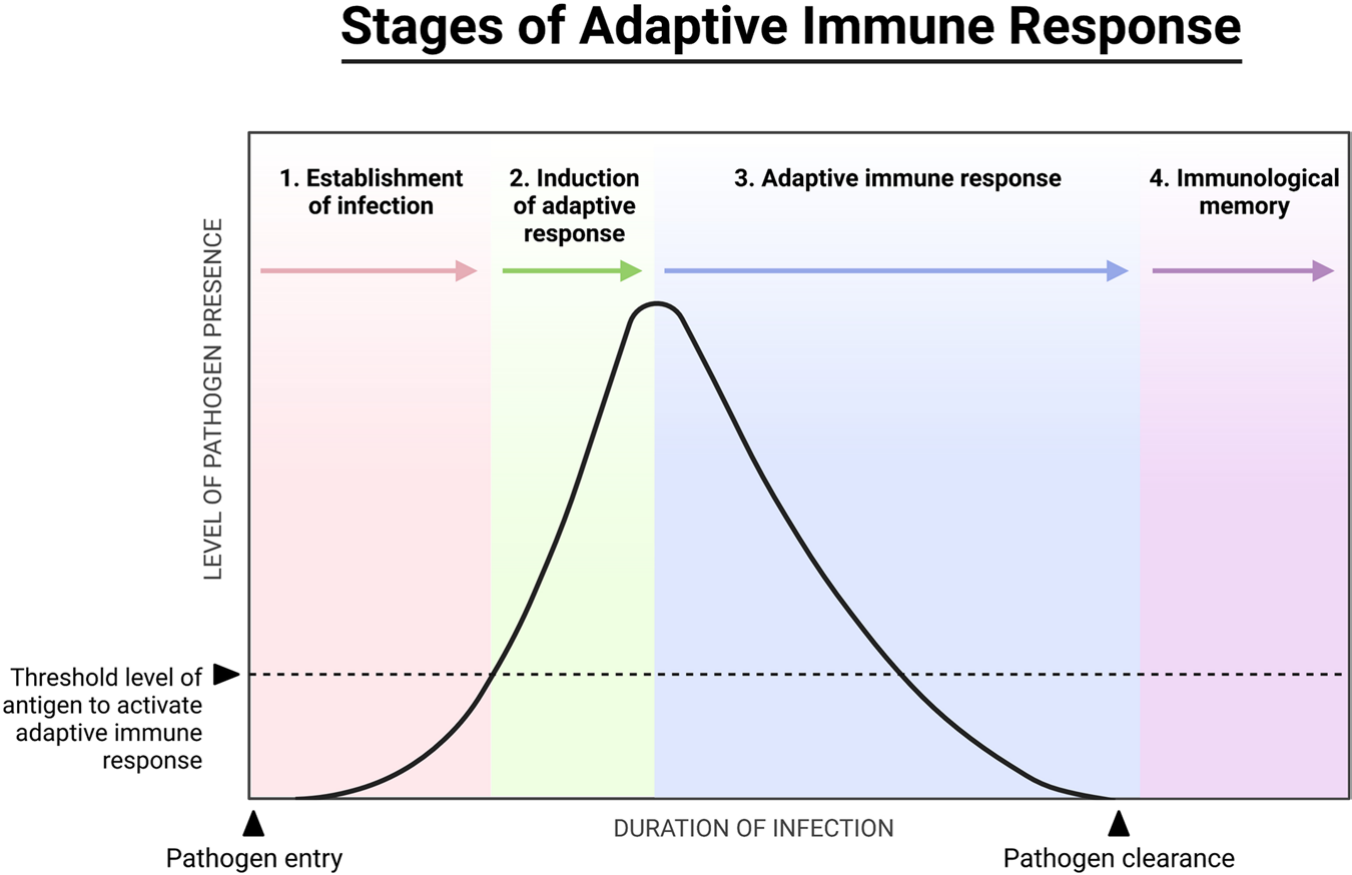
Stages of Adaptive Immune Response. This schematic view demonstrates the stages of infection (numbered), the level of pathogen presence in the host body, and the involvement of the adaptive immune response at each stage (black line).

The immune repertoire is a collection of trans-membrane proteins that assist with the recognition of different antigens and initiate an immune response. These proteins, located on the surface of T cells and known as T cell receptors (TCRs), are made of several chains (alpha through delta) and while sharing a general molecular structure, each TCR is slightly different. This repertoire has the potential to generate more than 10^15^ ^1^ different T cell receptors in humans. Once activated, the immune system sees major changes in its T cell composition; some T cells undergo clonal expansion^2^ and proliferation^3^, which later subsides. These dynamics govern the outcome of the immune response, and are key to understanding both the disease itself and its effective treatment^4^. Fig. 1 is a schematic view of the immune response in the face of infection, which also highlights the temporal nature of the response, rather than a binary state.

**Fig. 2:**
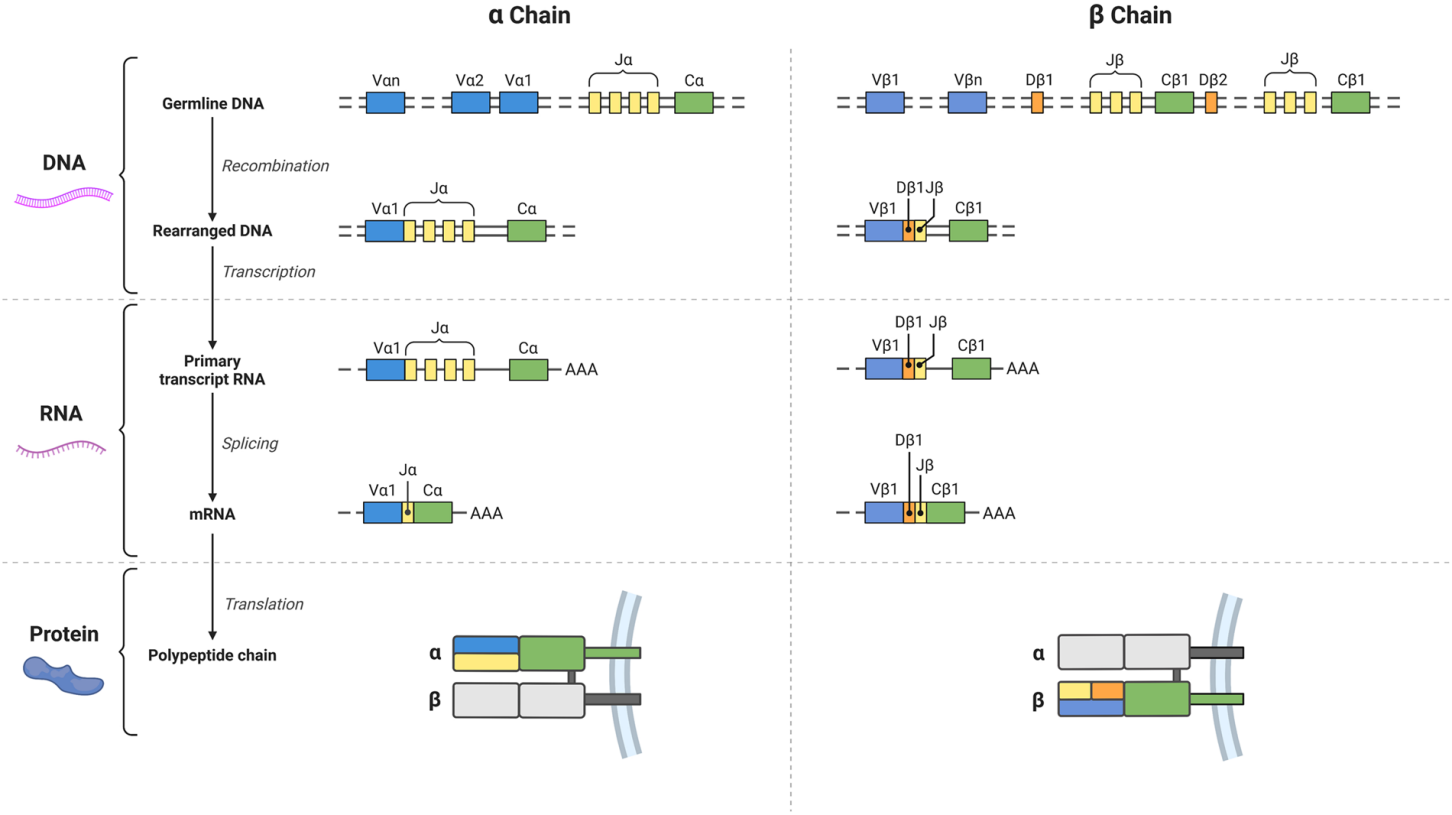
Schematic view describing the formation of alpha (left side) and beta (right side) chains of the TCR. These processes are collectively termed V(D)J recombination.

The immune system must face a varied list of potential threats; be it viruses, bacteria, or the body’s own malfunctioning cells. Recruitment and activation of immune cells is needed. For this to happen, the immune system must be diverse enough to interact with different antigens. This is achieved with three cellular and molecular mechanisms: 1) the recombination of the T cell alpha genes and beta genes by the RAG1–2 protein complex during T cell development, 2) the stochastic nature of the formation of the third complementarity-determining region (CDR3) of the T cell, and 3) the combination of alpha and beta chains. This entire process is termed V(D)J recombination^5,6^ (Fig. 2) and is key to the development of the adaptive immune system, and the correct formation of the rearranged receptors is critical to their association with  antigens.

Immune checkpoint inhibitors (“ICI”)^7–9^ are used as treatment for several types of cancer. This kind of treatment will suppress natural checkpoint and regulators of the immune system, resulting in an amplified immune response, one that is more likely to deal with cancerous cells. This type of treatment has recently been employed in colorectal cancer (“CRC”), with several positive results reported, specifically in metastatic CRC that is mismatch-repair-deficient and microsatellite instability-high (dMMR–MSI-H)^10^. The fundamental involvement of the immune response in this type of cancer makes ICI and its effect on the T cell repertoire especially important.

CTLA4 is a negative regulator of the immune response^11,12^, aimed at preventing hyperactivation and adjusting the intensity of the immune response by causing clonal anergy of lymphocytes. CTLA4 operates on several cellular mechanisms, such as CD28 antagonism in binding to B7 ligands and other co-stimulatory ligands, recruiting T cell inhibitory effectors, and preventing immune stimulating antibody conjugate^3^. Anti-CTLA4 will block all of the above, and mediate a stronger immune attack^13^.

Unlike the genetic code, the TCR repertoire is dynamic; its state, made readable with RNA sequencing, indicates the current status of the immune system. As such, several methods have been developed to quantify the TCR repertoire and its diverse components^14–16^, and many computational and analytical tools are constantly being developed and used to analyze these repertoires^17–19^. Many works have highlighted the importance of a diverse TCR repertoire for the immune system to perform optimally^1,20^, and while these works track and document changes to the repertoire, the supporting data is limited to pre- and post- treatment time points at best. Additionally, since a phenotypical change, such as reduction in tumor size, can occur some time after the peak of the immunological response, it can be difficult to create a snapshot of the immune state at the most relevant time. Moreover, the high variability in the favorable reaction of cancers to immunotherapy, makes it even challenging for researcher to quantify the true dynamics of the immune response^21^.

In light of the above, we designed and carried out an experiment aimed at creating a temporal profile of the dynamics of the immune response as it occurs. These data, made available here, would allow researchers from all backgrounds to observe the changes over time as they develop and eventually subside in response to treatment with anti-CTLA-4. Analyzing the behavior of the immune response, presented here in several forms, from raw reads to clonotype listings, would benefit efforts to better understand the immune response in cancer, and hopefully lead to better treatments.

## Methods

### Experimental procedure

**Fig. 3:**
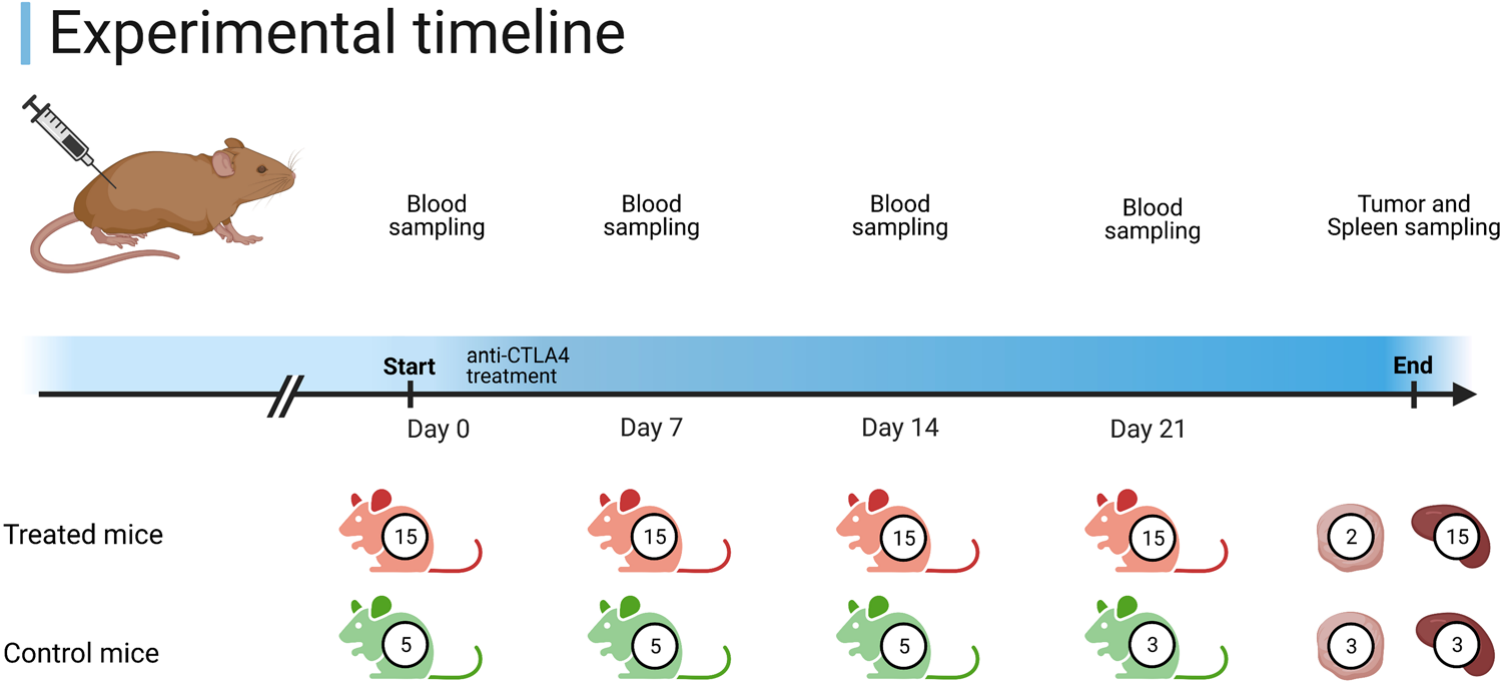
Experimental timeline. Treated mice (colored red) underwent injection of anti-CTLA-4 clone 9H10 on days 1, 3, and 6. Control mice (colored green) were injected with vehicle at similar times. Blood samples were taken on days 0, 7, 14 and 21. Tumor (when present) and spleen samples were collected at animal sacrifice at the end of the experiment. Numbers indicate the number of mice viable for sampling.

In this experiment we used the mouse tumor cell line MC38, a grade III mouse colon adenocarcinoma, induce in a syngeneic mouse model. An estimated 5·10^5^ MC38 cells were subcutaneously implanted to immunocompetent C57BL/6 N mice of the C57Bl/6NAnNCrl strain. The untreated group (5 mice) was dosed with 10 ml/kg/day of vehicle on day 1, 3 and 6 of the experiment. The treated group (15 mice) was dosed with the immune checkpoint inhibitor antibody anti-mCTLA4 (5/2.5 mg/kg/day at 1/3, 6). Following treatment, blood samples for the isolation of PBMCs were taken at days 0, 7, 14, and 21. The experimental timeline is shown in Fig. 3.

### Animal health and ethics

All experiments and protocols were approved by the animal welfare body at CR Discovery Research Services Germany and the local authorities, and were conducted according to all applicable international, national and local laws and guidelines. Only animals with unobjectionable health were selected to enter testing procedures. The animals were delivered at the age of four to six weeks and were used for experiments after at least one week of acclimatization. Animals were arbitrarily numbered during tumor implantation using radio frequency identification transponders. Each cage was labeled with a record card indicating all relevant experimental details.

### Animal housing conditions

Animals were housed in individually ventilated type II and III cages, based on group size, and were kept under a 14 L:10D artificial light cycle. The temperature inside the cages was maintained at 25 °C with a relative humidity of 40–70% and 60–65 air changes/hour in the cage. Dust-free bedding consisting of aspen wood chips with approximate dimensions of 5 × 5 × 1 mm and additional nesting material was used. The cages, bedding, and the nesting material were changed weekly. The animals were fed autoclaved Teklad Global 19% Protein Extruded Diet (T.2019S.12) from Envigo RMS SARL and had access to sterile filtered and acidified (pH 2.5) tap water that was changed per week. Feed and water were provided ad libitum. All materials were autoclaved prior to use.

### Immunotherapy treatment

A dosing solution with a concentration of 0.5 mg/ml for dosing at 5 mg/kg/day was freshly prepared on dosing day 1 by diluting 203 *μ*L of anti-mCTLA4 stock solution with 2797 *μ*L of PBS. A dosing solution with a concentration of 0.25 mg/ml for dosing at 2.5 mg/kg/day was freshly prepared on dosing day 3 and 6 by diluting 101 *μ*L of anti-mCTLA4 with 2899 *μ*L of PBS. All dosing solutions were administered in a dose volume of 10 ml/kg.

### PBMCs purification from blood samples

100*μ*L of blood was collected by retro-orbital sinus puncture under isoflurane anesthesia. PBMCs were prepared from 100 *μ*L blood by spinning down cells and lysing pellets with ACK lysis buffer (150 mM ammonium chloride, 10 mM potassium bicarbonate, 0.1 mM EDTA, pH 7.2–7.4) to remove erythrocytes. The resulting PBMC were frozen in 10% DMSO/90% FCS at 80 °C until shipment.

### Solid sample collection

Tumors (where still visible) were collected immediately after euthanasia, and directly transferred to liquid nitrogen (snap-frozen samples). Spleens were collected immediately after euthanasia, and directly transferred to liquid nitrogen (snap-frozen samples).

### RNA purification from blood, tumor and spleen samples

PBMCs samples were thawed, and their total RNA content extracted using the RNeasy mini kit (Qiagen). Tumor and spleen samples were thawed, weighted and applied to Trizol (Invitrogen) purification procedure according to the manufacturer protocol. The obtained RNA product was then evaluated for its integrity value (RIN) to better standardize RNA quality. Next, RNA concentrations were determined by qubit, which provides an accurate method for the quantitation of low abundance RNA samples.

### Library preparation

A fixed total RNA concentration of 250 ng from each sample was subjected to the SMARTer TCR profiling kit (Takara); a single cDNA strand is synthesized at the first round of reverse transcription, using poly T primer for the 3’ end and a smarter oligo (Switching Mechanism at 5’ End of RNA Template) for the 5’ ends, catalyzed by MMLV-derived SMARTScibe Reverse Transcriptase. By employing 5’ RACE-like approach, the SMARTer TCR profiling kit allows capturing complete V(D)J variable regions of TCR alpha and beta chains and in the following step, each fragment was exponentially increased by PCR. On each dsDNA fragment, dedicated adapters containing flow cell-binding sites, P5 and P7, were inserted to allow the enriched library fragments to attach to the cell flow surface. The TCR sequencing library was size-selected and purified using AMPure XP beads. The generated libraries were measured for their DNA concentration by qubit and assessed for their size by Tapestation or Bioanalyzer.

### Sequencing and reads processing

TCR sequencing was performed on an Illumina Miseq sequencer using the 600-cycle Miseq reagent kit v3 (Illumina) with pair-end, 2 × 300 base pair reads. Library pool molarity was 4 nM. Transforming raw reads to alpha and beta CDR3 sequences was done by using MiXCR, a universal framework that processes big immunome data from raw sequences and output quantitated clonotypes (Bolotin *et al*.^18^). For the alpha chain we obtained 13·10^6^ reads with an average of 171628 copies and 25159 unique amino-acid clones, and for the beta chain we obtained 18.6·10^6^ with an average of 245867 copies and 49725 unique amino-acid clones. In order to avoid bias originating from different sample sizes, we sub-sampled 97048 sequences from each sample, as the number of clones in the smallest sample.

### Randomization and blinding

The initial division of treatment (15 mice, labeled F to T) and control groups (5 mice, labeled A to E) was done randomly. At the end of the experiment, each mouse was assigned to a response group according to the changes to its tumor size in response to treatment: Early responders (mice L, N, P, Q, R) – mice that showed early response to treatment and reduction in tumor volume; Mid-responders – mice that showed medium response to treatment and reduction in tumor volume (mice J, M, O, S, T), and Late responders - mice that showed later response to treatment and reduction in tumor volume (mice F, G, H, I, K).

### Clonotyping

To generate the alpha and beta chains clonotyping lists, the tool MiXCR was used^18^. MiXCR is a universal framework that processes big immunome data from raw sequences to quantitated clonotypes. MiXCR was used with the following arguments: −−*speciesmmu*−−*libraryde f ault* −−*threads*12−−*reportreport* − *prna*−*seq*−*OvParameters.geneFeatureToAlign* = *VTranscriptWithP*−*OvParameters*.*parameters*. *f loatingLe f tBound* = true−*OjParameters.parameters. f loatingRightBound* = *f alse*−*OcParameters.parameters*. *f loatingRightBound* = *true*. Sample code for similar use is provided in the code availability section of this work.

## Data Records

The TCR-Seq data were deposited in the NCBI Sequence Read Archive (SRA) under the accession number PRJNA658348^22^. This data appears as zipped FASTQ files, as is standard for next generation sequencing results. This data represents the TCR sequencing of all data points: blood samples taken on days 0, 7, 14, and 21 from treated and control mice, as well as TCR sequencing of tumors and spleens taken at the end of the experiment.

The Alpha and Beta chain TCRs were deposited on FigShare under the following 10.6084/m9.figshare.19287506^23^ (alpha) and 10.6084/m9.figshare.19287512^24^ (beta). These datasets appear as tabular text files, and include information on each clonotype in the data. This includes a unique clone ID, clone count, the clone’s fraction out of the total number of clones, the actual clonal sequence in base-pairs, and more. For complete details of the fields populating this data, we suggest reading the documentation at https://mixcr.readthedocs.io/en/master/export.html. Note that this table is sorted according to the clone count by default, with the most prevalent clonotypes appearing on top.

The metadata for each clonotype dataset were deposited on FigShare under the following 10.6084/m9.figshare.19312262^25^ (alpha) and 10.6084/m9.figshare.19312265^26^ (beta). These data sets appear as tabular text files, and include meta-information on each data point. This includes the sample name (matching the RNA-Seq data files), the sample ID, Time point, a mouse identifier, the group (either treated or control), the tissue from which the sample was taken (Blood, Tumor or Spleen), and how quickly, if at all, a given mouse responded to treatment (as determined by tumor size measurements throughout the experiment)

**Fig. 4:**
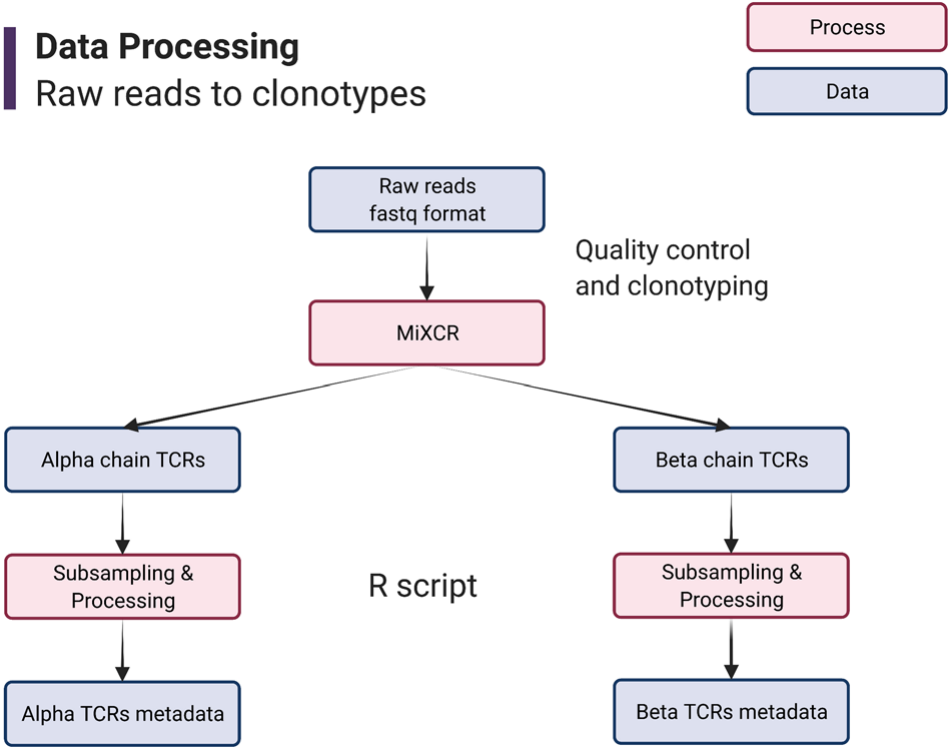
Data processing flow chart. Pink rectangles indicate processes used to generate data, blue rectangles indicate data sets available in this work.

The entire data collection process, as well as the data made available in this work, is presented as a work flow in Fig. 4.

## Technical Validation

### TCR-seq validation

To validate the raw sequencing data, we used FASTQC^27^, A quality control tool for high throughput sequence data. Results showed that all sequences had high per sequence quality scores”. Additionally, all samples had valid percentage of base calls at each position for which an N was called, and all samples were of the same length of 301 basepairs, as expected. It should be noted that as these sequences are of T cell receptors only, and do not align a standard mouse genome, as is evident in the low alignment score of all samples, as well as the presence of promoters in the data.

### Clonotyping validation

**Fig. 5:**
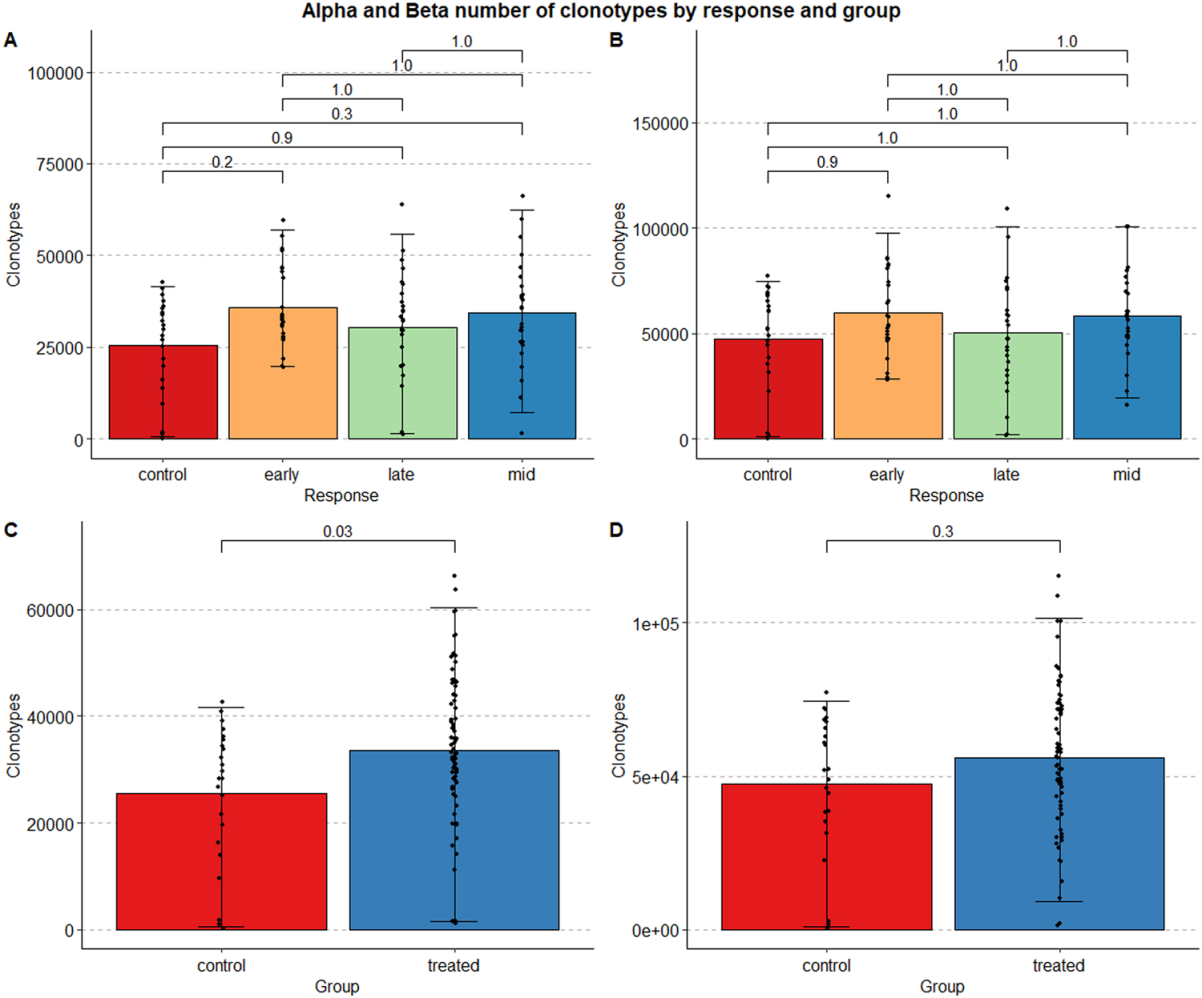
Alpha and Beta number of clonotypes by response and group. Panels on the left (**A**,**C**) indicate the distribution of the number of clonotypes belonging to the Alpha chain of the TCR, colored by response (top row) and treatment group (bottom row). Panels on the right (**B**,**D**) indicate the distribution of the number of clonotypes belonging to the Beta chain of the TCR, in a similar manner.

To demonstrate that the clonotype data is valid, we used Immunarch^28^ to measure the number and length of the quantified clonotypes for each chain. As can be seen in Fig. 5, there is no significant difference in number of clonotypes among between the treatment groups, nor is there a significant difference between the number of clonotypes between the response groups.

**Fig. 6:**
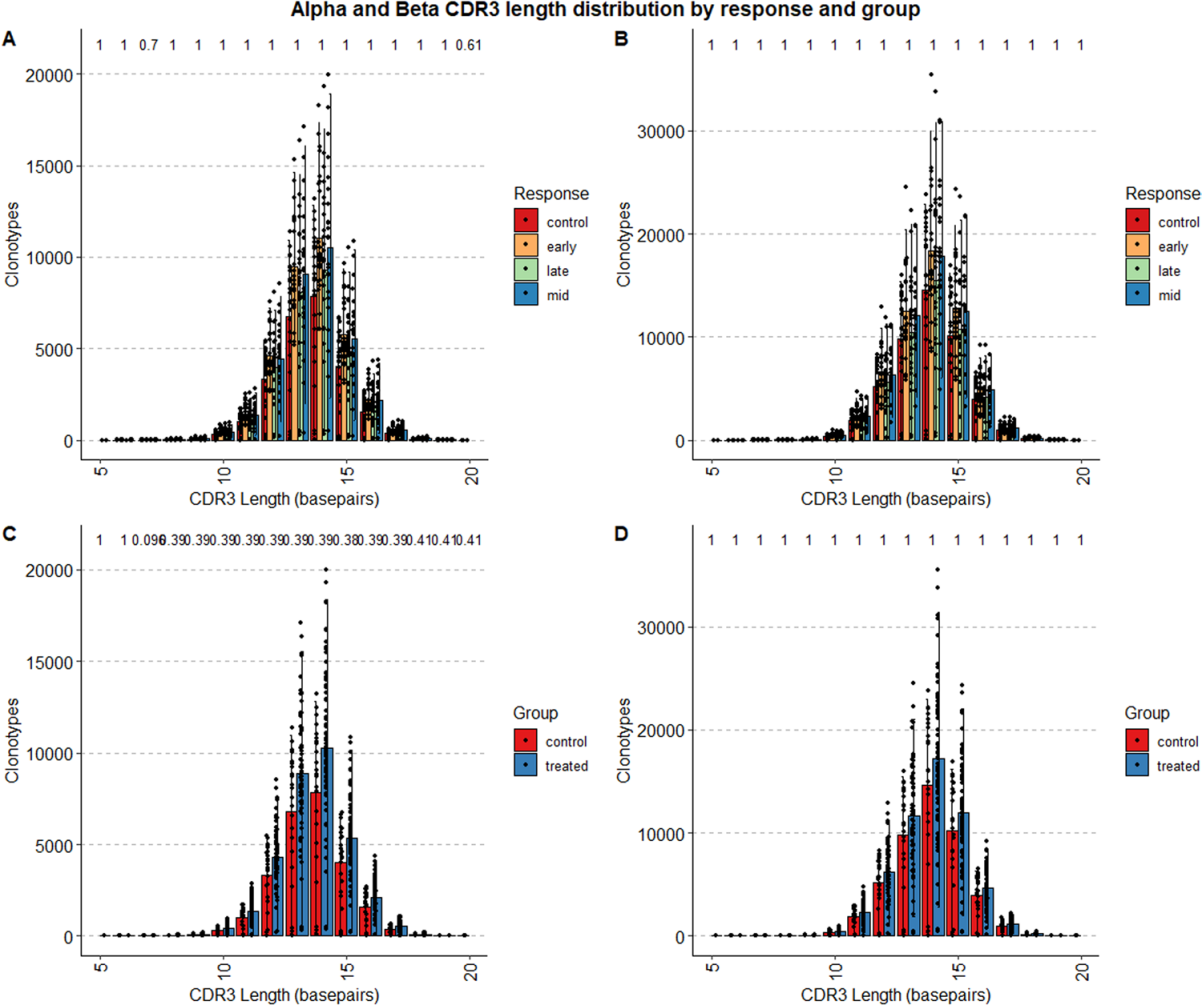
Alpha and Beta CDR3 length distribution by response and group. Panels on the left (**A**,**C**) indicate the distribution of the length of the CDR3, in base-pairs, belonging to the Alpha chain of the TCR, colored by response (top row) and treatment group (bottom row). Panels on the right (**B**,**D**) indicate the distribution of the length of the CDR3, in base-pairs, belonging to the Beta chain of the TCR, in a similar manner.

Similarly, as can be seen in Fig. 6, there is no significant difference in the length of the CDR3 section of the clonotypes between the treatment groups, nor is there a significant difference in the length of the CDR3 section of the clonotypes between the response groups.

The above emphasizes the validity of the data, and allows for unbiased comparison between different treatment or response groups.

## Usage Notes

Reuse of the data provided herein is possible either by re-construction of the clonotypes from the raw sequencing files, or using our built clonotyping list and continue the analysis from there. Users should note that when constructing their own clonotyping data, different arguments can be used to obtain fields of interest in the data.

The raw repertoire sequencing data could be used in several ways; first, clonotype quantification can be performed in several ways and under different conditions^29^. We recommend the use of MiXCR (see code examples section for our application), but other tools such as IgBLAST^30^ and ImRep (https://github.com/mandricigor/imrep) have useful features. Alternatively, the data can be used like any other short-read RNA sequencing data, although generated from T cells only; such a work-flow would include alignment, assembly and quantification of gene expression. While tools for each step exist, we recommend using tools able to perform all of these steps, as exemplified in a recent review by Stark *et al*.^31^.

Once clonotyping is performed, these data, both of the alpha and beta chains, could be used with many existing, specialized pipelines. These include Immunarch^28^, Immunoseq analyzer^32^, Immcantation Framework^33^ and VDJtools^17^. A recent useful review by Arunkumar *et al*. compares these tools and others^19^. Analysis such as this can be done to estimate the number of copies a specific clonotype has, compare clonotypes to those found in external databases, or attempt to tie the presence of clonotypes with response to treatment, or lack thereof.

Finally, the data presented here could be used with machine learning approaches to predict response to treatment. This could be done either with dedicated platforms such as ImmuneML^34^, or with any other tool or approach that could uncover a connection between response to treatment and the composition of the repertoire. Any such methods would benefit from the temporal nature of the data, and could support approaches dedicated to precise treatment of individual patients. We would recommend the use of time-series analysis to the data presented here.

## Data Availability

Several scripts are made available to allow users of all backgrounds and experience levels to access this data. After downloading the raw sequencing data, users can use the following code^35^ to perform clonotyping and obtain a list of alpha and beta chains from each sample, as well as a report file with details on the success of the process. Note that the user should have a recent version of MiXCR installed, as detailed in the^18^ MiXCR documentation. When using the clonotype data as input, either after following the step above or by downloading the clonotyping data made available here, the user can use an R script^36^ to gather some statistics and perform an initial analysis of the clonotypes. Note that the user should have a recent version of the coding language R installed, and the appropriate packages up to date. The code also demonstrates performing sampling of the data, to prevent bias caused by samples with a larger number of clonotypes.

## References

[1] Nikolich-Žugich, J., Slifka, M. K. & Messaoudi, I. The many important facets of T-cell repertoire diversity. *Nat. Rev. Immunol*. **4**, 123–132. URL https://www.nature.com/articles/nri1292. 10.1038/nri1292. Number: 2 Publisher: Nature Publishing Group (2004). 10.1038/nri1292 15040585

[2] Adams, N. M., Grassmann, S. & Sun, J. C. Clonal expansion of innate and adaptive lymphocytes. *Nat. Rev. Immunol*. **20**, 694–707. Publisher: Nature Publishing Group (2020). 10.1038/s41577-020-0307-4 PMC13119617 32424244

[3] Heinzel, S., Marchingo, J. M., Horton, M. B. & Hodgkin, P. D. The regulation of lymphocyte activation and proliferation. *Curr. Opin. Immunol*. **51**, 32–38 URL https://www.sciencedirect.com/science/article/pii/S0952791517300948. 10.1016/j.coi.2018.01.002 (2018). 10.1016/j.coi.2018.01.002 29414529

[4] Dash, P. *et al*. Quantifiable predictive features define epitope-specific T cell receptor repertoires. *Nat*. **547**, 89–93. URL https://www.nature.com/articles/nature22383. 10.1038/nature22383. Number: 7661 Publisher: Nature Publishing Group (2017). 10.1038/nature22383 PMC5616171 28636592

[5] Chi, X., Li, Y. & Qiu, X. V(D)J recombination, somatic hypermutation and class switch recombination of immunoglobulins: mechanism and regulation. *Immunol*. **160**, 233–247 URL https://onlinelibrary.wiley.com/doi/abs/10.1111/imm.13176. 10.1111/imm.13176. _eprint: (2020). 10.1111/imm.13176 PMC7341547 32031242

[6] Alt, F. W. *et al*. VDJ recombination. *Immunol. Today* **13**, 306–314. URL https://www.sciencedirect.com/science/article/pii/0167569992900437. 10.1016/0167-5699(92)90043-7 (1992). 10.1016/0167-5699(92)90043-7 1510813

[7] Darvin, P., Toor, S. M., Sasidharan Nair, V. & Elkord, E. Immune checkpoint inhibitors: recent progress and potential biomarkers. *Exp. & Mol. Medicine* **50**, 1–11. URL https://www.nature.com/articles/s12276-018-0191-1. 10.1038/s12276-018-0191-1. Number: 12 Publisher: Nature Publishing Group (2018). 10.1038/s12276-018-0191-1 PMC6292890 30546008

[8] Esfahani, K. *et al*. A Review of Cancer Immunotherapy: From the Past, to the Present, to the Future. *Curr. Oncol*. **27**, 87–97. URL https://www.mdpi.com/1718-7729/27/12/5223. 10.3747/co.27.5223. Number: s2 Publisher: Multidisciplinary Digital Publishing Institute (2020).

[9] Kidman, J. *et al*. Characteristics of TCR Repertoire Associated With Successful Immune Checkpoint Therapy Responses. *Front. Immunol*. **11**. URL https://www.frontiersin.org/article/10.3389/fimmu.2020.587014 (2020). 10.3389/fimmu.2020.587014 PMC7591700 33163002

[10] Ganesh, K. *et al*. Immunotherapy in colorectal cancer: rationale, challenges and potential. *Nat. Rev. Gastroenterol. & Hepatol*. **16**, 361–375. URL https://www.nature.com/articles/s41575-019-0126-x. 10.1038/s41575-019-0126-x. Number: 6 Publisher: Nature Publishing Group (2019). 10.1038/s41575-019-0126-x PMC7295073 30886395

[11] Chae, Y. K. *et al*. Current landscape and future of dual anti-CTLA4 and PD-1/PD-L1 blockade immunotherapy in cancer; lessons learned from clinical trials with melanoma and non-small cell lung cancer (NSCLC). *J. for ImmunoTherapy Cancer* **6**, 39. URL 10.1186/s40425-018-0349-3 (2018). 10.1186/s40425-018-0349-3 PMC5956851 29769148

[12] Leach, D. R., Krummel, M. F. & Allison, J. P. Enhancement of Antitumor Immunity by CTLA-4 Blockade. *Sci*. **271**, 1734–1736. URL https://www.science.org/doi/abs/10.1126/science.271.5256.1734. 10.1126/science.271.5256.1734. Publisher: American Association for the Advancement of Science (1996). 10.1126/science.271.5256.1734 8596936

[13] Waldman, A. D., Fritz, J. M. & Lenardo, M. J. A guide to cancer immunotherapy: from T cell basic science to clinical practice. *Nat. Rev. Immunol*. **20**, 651–668. URL https://www.nature.com/articles/s41577-020-0306-5. 10.1038/s41577-020-0306-5. Number: 11 Publisher: Nature Publishing Group (2020). 10.1038/s41577-020-0306-5 PMC7238960 32433532

[14] Benichou J, Ben-Hamo R, Louzoun Y, Efroni S (2012). Rep-Seq: uncovering the immunological repertoire through next-generation sequencing. Immunol..

[15] Warren, R. L. *et al*. Exhaustive T-cell repertoire sequencing of human peripheral blood samples reveals signatures of antigen selection and a directly measured repertoire size of at least 1 million clonotypes. *Genome Res*. **21**, 790–797. URL https://genome.cshlp.org/content/21/5/790. 10.1101/gr.115428.110. Company: Cold Spring Harbor Laboratory Press Distributor: Cold Spring Harbor Laboratory Press Institution: Cold Spring Harbor Laboratory Press Label: Cold Spring Harbor Laboratory Press Publisher: Cold Spring Harbor Lab (2011). 10.1101/gr.115428.110 PMC3083096 21349924

[16] Georgiou, G. *et al*. The promise and challenge of high-throughput sequencing of the antibody repertoire. *Nat. Biotechnol*. **32**, 158–168. URL https://www.nature.com/articles/nbt.2782. 10.1038/nbt.2782. Number: 2 Publisher: Nature Publishing Group (2014). 10.1038/nbt.2782 PMC4113560 24441474

[17] Shugay, M. *et al*. VDJtools: Unifying Post-analysis of T Cell Receptor Repertoires. *PLOS Comput. Biol*. **11**, e1004503. URL https://journals.plos.org/ploscompbiol/article?id=10.1371/journal.pcbi.1004503. 10.1371/journal.pcbi.1004503. Publisher: Public Library of Science (2015). 10.1371/journal.pcbi.1004503 PMC4659587 26606115

[18] Bolotin DA (2015). MiXCR: software for comprehensive adaptive immunity profiling. Nat. Methods.

[19] Arunkumar, M. & Zielinski, C. E. T-Cell Receptor Repertoire Analysis with Computational Tools—An Immunologist’s Perspective. *Cells* **10**, 3582. URL https://www.mdpi.com/2073-4409/10/12/3582. 10.3390/cells10123582. Number: 12 Publisher: Multidisciplinary Digital Publishing Institute (2021). 10.3390/cells10123582 PMC8700004 34944090

[20] Robins, H. Immunosequencing: applications of immune repertoire deep sequencing. *Curr. Opin. Immunol*. **25**, 646–652. URL https://www.sciencedirect.com/science/article/pii/S0952791513001520. 10.1016/j.coi.2013.09.017 (2013). 10.1016/j.coi.2013.09.017 24140071

[21] Philip, H. *et al*. A T cell repertoire timestamp is at the core of responsiveness to CTLA-4 blockade. *iScience* **24**, 102100. URL https://www.sciencedirect.com/science/article/pii/S2589004221000687. 10.1016/j.isci.2021.102100 (2021). 10.1016/j.isci.2021.102100 PMC7876555 33604527

[22] (2023). NCBI SRA.

[23] (2022). figshare.

[24] (2022). figshare.

[25] (2022). figshare.

[26] (2022). figshare.

[27] Babraham Bioinformatics - FastQC A Quality Control tool for High Throughput Sequence Data. URL https://www.bioinformatics.babraham.ac.uk/projects/fastqc/.

[28] Popov A (2022). Zenodo.

[29] Miho, E. *et al*. Computational Strategies for Dissecting the High-Dimensional Complexity of Adaptive Immune Repertoires. *Front. Immunol*. **9**. URL https://www.frontiersin.org/article/10.3389/fimmu.2018.00224 (2018). 10.3389/fimmu.2018.00224 PMC5826328 29515569

[30] Ye, J., Ma, N., Madden, T. L. & Ostell, J.M. IgBLAST: an immunoglobulin variable domain sequence analysis tool. *Nucleic Acids Res*. **41**, W34–W40. URL https://www.ncbi.nlm.nih.gov/pmc/articles/PMC3692102/. 10.1093/nar/gkt382 (2013). 10.1093/nar/gkt382 PMC3692102 23671333

[31] Stark, R., Grzelak, M. & Hadfield, J. RNA sequencing: the teenage years. *Nat. Rev. Genet*. **20**, 631–656. URL https://www.nature.com/articles/s41576-019-0150-2. 10.1038/s41576-019-0150-2. Number: 11 Publisher: Nature Publishing Group (2019). 10.1038/s41576-019-0150-2 31341269

[32] Morin, A. *et al*. Immunoseq: the identification of functionally relevant variants through targeted capture and sequencing of active regulatory regions in human immune cells. *BMC Med. Genomics* **9**, 59. URL 10.1186/s12920-016-0220-7 (2016). 10.1186/s12920-016-0220-7 PMC5022205 27624058

[33] Vander Heiden, J. A. *et al*. pRESTO: a toolkit for processing high-throughput sequencing raw reads of lymphocyte receptor repertoires. *Bioinforma*. **30**, 1930–1932. URL 10.1093/bioinformatics/btu138 (2014). 10.1093/bioinformatics/btu138 PMC4071206 24618469

[34] Pavlović, M. *et al*. The immuneML ecosystem for machine learning analysis of adaptive immune receptor repertoires. *Nat. Mach. Intell*. **3**, 936–944. URL https://www.nature.com/articles/s42256-021-00413-z. 10.1038/s42256-021-00413-z. Number: 11 Publisher: Nature Publishing Group (2021). 10.1038/s42256-021-00413-z PMC10312379 37396030

[35] (2022). figshare.

[36] (2022). figshare.

